# Finite Element Analysis of Impact for Helmeted and Non-helmeted Head

**DOI:** 10.1007/s40846-017-0324-3

**Published:** 2017-09-25

**Authors:** Ievgen Levadnyi, Jan Awrejcewicz, Yan Zhang, Márcio Fagundes Goethel, Yaodong Gu

**Affiliations:** 1 Department of Automation, Biomechanics and Mechatronics, Lodz University of Technology, 1/15 Stefanowski Str, 90–924 Lodz, Poland; 2 Institute of Vehicles, Warsaw University of Technology, 84 Narbutta Str, 02–524 Warsaw, Poland; 3 Faculty of Sports Science, Ningbo University, Ningbo, China; 4 Research Academy of Grand Health Interdisciplinary, Ningbo University, 818 Fenghua Road, Ningbo, 315211 China

**Keywords:** Head injury, Impact severity, Protective helmet, FEM

## Abstract

This study investigated the influence of human head impact on the severity of traumatic brain injury. Simulation of the dynamic impact of a human head was performed using FEM (finite element method) and employing HIC (Head Injury Criterion). The study of traumatic brain injury included impacts with the occiput, temporal, forehead, and parietal part of the head, and the impact velocity at the surface ranged from 1 to 7 m/s. The following characteristics were considered and analyzed in the simulation: duration of the impact, intracranial pressure, HIC, and change in accelerations at the center of gravity of the brain. The computed distribution of pressure values in the brain during an impact confirmed the theory of inertial intracranial brain displacement. The effect of a protective helmet aimed at reducing the severity of traumatic brain injury was investigated, and a method to determine rational helmet parameters was developed. In the case of the protected head, impact acceleration occurred over a longer period of time, which yielded a reduction in the brain load compared to the unprotected head. The developed method allows us to predict the severity of traumatic brain injury (TBI) in the protected/unprotected human head and to provide recommendations for the determination of rational parameters for manufacturing personal protective equipment for the head.

## Introduction

Traumatic brain injury (TBI) includes damage to the skull, brain, blood vessels, nerves, and meninges. TBI is a leading cause of injury death and disability, especially among men from 20 to 40 years old. According to the World Health Organization (WHO), there are more than ten million people suffering from TBI each year worldwide, out of which 200–300 thousand cases result in death [1–3].

TBI occurs in the case of a moving head crashing into a stationary rigid object instantaneously (approximately 50 ms). It is also observed that TBI occurs when a rapidly moving head abruptly changes direction without striking [4]. A traumatic effect is associated with two main destructive factors: contact and inertia [5–7]. Contact factor is characterized by local damage of the scalp, skull and brain. Inertia factor is characterized by acceleration or deceleration of the head and the elements of its structure. In the head, positive pressure (coup injury) shows on the side of impact with an object, while negative pressure (countercoup injury) shows on the opposite side. If the acceleration/deceleration increases dramatically, coup and countercoup injury can lead to brain damage, which is the case when the brain comes into contact and interacts with bony protrusions of the skull.

By following recommendations, the possibility of TBI can be effectively reduced. For example, wearing a helmet is the single most effective way of reducing head injuries and fatalities that result from traffic accidents involving bicycles [8, 9] or motorcyclists [10, 11], dangerous workplaces [12, 13], and dangerous sports [14, 15]. Mathematical modeling using finite element method (FEM) has obvious advantages, such as the low cost of numerical experiments with the ability to change basic parameters, such as material properties, impact velocity, and side of head impact, and it allows us to gain a large range of results that are difficult to measure non-invasively with equipment, including pressure, acceleration, velocity, stress–strain, and force magnitudes. It is important to predict the impact’s effect on the head to improve the principles of diagnosis and treatment of TBI. Despite the fact that modeling the human head using FEM has been intensively developed in the last ten years [16–22], it is still far from being able to explain the mechanisms of brain damage and predicting the consequences of TBI. Issues, such as the rheology of biomaterials, mechanical interaction of the brain and skull surface, the influence of voids on stress distribution, and the consideration of a multi-layer structure of the brain, should be taken into account to obtain a valuable and validated impact model.

The majority of studies focused on investigating the occurrence of brain damage do not consider the area between the hitting surface of the object and the head, head position, direction and angle of impact, location force application, anatomical structure and protective function aimed at the reduction the force of impact. Compared to a two-dimensional (2D) model of the human head [23, 24], a three dimensional (3D) head model could provide realistic information on mechanical impacts.

The purpose of this study is to investigate the impact of a 3D model of the human head in cases in which the impact was on occiput, temporal, forehead, and parietal part of the head and the impact velocity ranged from 1 to 7 m/s. In addition, the effect of a protective helmet to reduce the severity of traumatic brain injury has also been investigated. The most efficient parameters of a protective helmet were chosen based on the simulations of head impacts in the helmets. In this case, the thickness value of the protective foam varied from 2 to 20 mm in increments of 2 mm, and the thickness of the top protective shell varied from 1 to 10 mm in increments of 1 mm. To achieve a real TBI model, the basic parameters of impact included the duration of impact, intracranial pressure, head injury criterion (HIC), change acceleration of the brain under different impact loads and the presence and absence of a protective helmet.

## Materials and Methods

### Brain and Skull

**Fig. 1:**
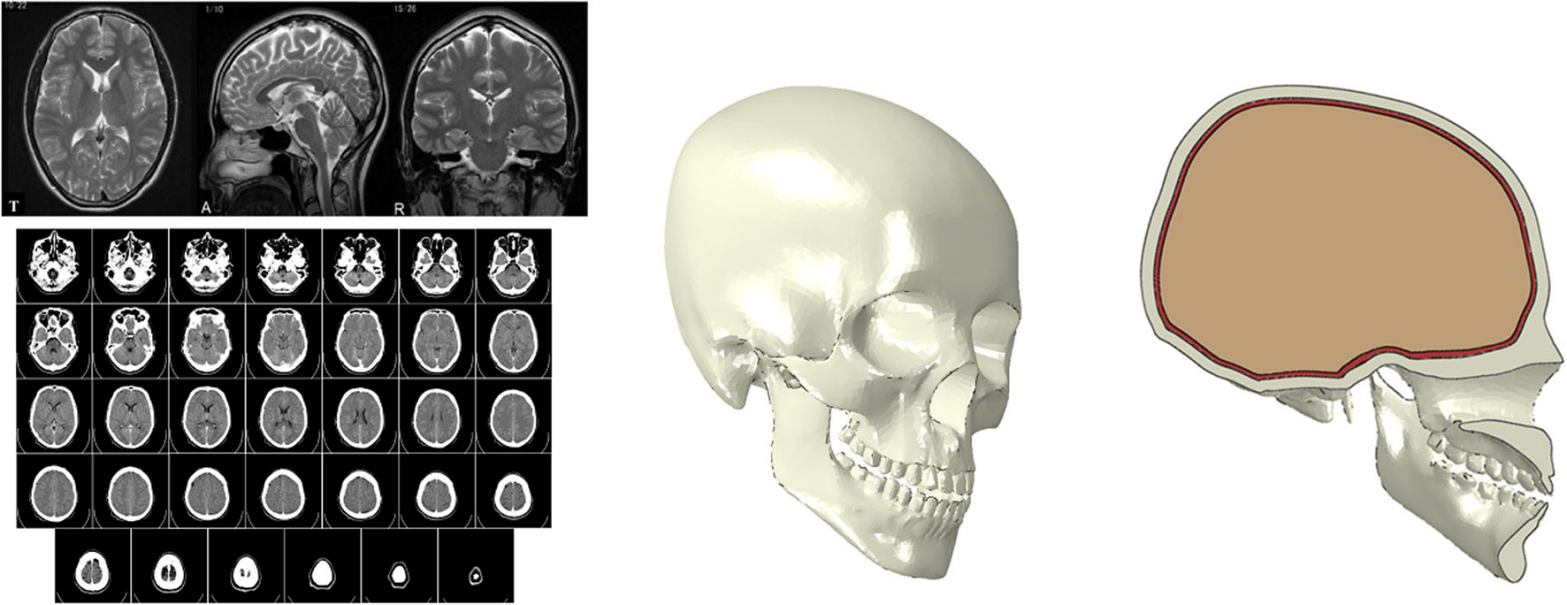
Three-dimensional geometric models of the brain and skull created using computed tomography images

To conduct a qualitative numerical analysis and obtain reliable results, it is necessary to ensure similarity in the behavior of the developed model and a real human head. The magnetic resonance imaging (MRI) technique is currently one of the main methods for diagnostics and the construction of 3D models of numerous biomechanical objects (Fig. 1). The algorithm to generate the skull-brain a three-dimensional geometric model included several steps. At first, a set of two-dimensional MRI images of an actual human head of a patient were downloaded for subsequent segmentation of the object. The segmentation was performed based on the obtained axial projections of the object, using the selection as a separate mask. The MRI data were processed using MIMICS (Materialise, N.V.—Belgium) software. Then, the stereolithography (STL) file with the 3D object made on a mask was created. In the third step, the quality of the model was improved by employing various surface smoothing functions. For finite element analysis in the present study, the three-dimensional geometric model of a human head was imported and meshed using ABAQUS software (version 6.14, Dassault Systems, 2015). The average approximate characteristic length of the elements was equal to 3 mm. The whole finite element human head model consisted of 607,310 tetrahedral elements and approximately 523,438 nodes.

The next stage of the modeling was to add material properties for different structures of the head. The material property of the brain tissue is assumed to be isotropic and linear viscoelastic with shear relaxation behavior, as described by the following equation:

1 $$G(t) = G_{\infty } + (G_{0} - G_{\infty } )e^{{ - \beta {\text{t}}}} ,$$

where *G*(t)—long-time (infinite) shear modulus, G_0_—short-time shear modulus, *β*—decay coefficient, and t—time. The material properties are taken from [25]: bulk modulus = 1125 MPa, short time shear modulus = 0.049 MPa, long time shear modulus = 0.0167 MPa, decay constant = 145 s^−1^, and density ρ = 1040 kg/m^3^.

The brain-skull interface has been modeled using the cerebra-spinal fluid (CSF) approach. The CSF bathes the brain, maintaining a uniform pressure within the cranium in a normal head and performing an important function in protecting the brain from jolts that would cause it to hit the bony walls of the cranium. An average CSF thickness of approximately 2 mm was used, which corresponds to approximately 120–150 ml of subdural and subarachnoidal CSF [26]. The mechanical behavior of CSF is assumed to be linear elastic with a low shear modulus. In this case, the element loses its ability to support shear stress, and only compressive hydrostatic stress states are possible. The following parameters [26] in our study are fixed: elastic modulus E = 12,000 (Pa); Poisson’s ratio ν = 0.49; and density ρ = 1130 (kg/m^3^). Because CSF does not constrain the brain from motion relative to the skull, sliding contact definitions were used for these interfaces [27]. The behavior of bone material for the skull bone has been chosen as isotropic elastic–plastic with the following properties [17]: elastic modulus E = 8000 MPa, Poisson ratio ν = 0.22, density ρ = 1200 kg/m^3^, and ultimate compression/tensile stress σ_BC_ = 145 MPa/σ_BT_ = 90 MPa.

At the skull-rigid surface interface, a friction coefficient of 0.4 has been employed.

### Helmet

The second finite element model was used to evaluate the effect of a protective helmet in reducing the severity of traumatic brain injury. For simulation, sketches were first made of the helmet to obtain its dimensions. Then, CAD software Solidworks 2015 was used to model the safety helmet. The role of the helmet as personal protective equipment (PPE) is aimed at damping the impact energy and providing safety to the brain and skull from forces that would have occurred in the case of an impact without a helmet. The most efficient parameters of the protective helmet were chosen based on simulations of head impacts in the helmets. In this case, the thickness value of the protective foam varied from 2 to 20 mm in increments of 2 mm, and the thickness of the top protective shell varied from 1 to 10 mm in increments of 1 mm.

The behavior of foam material was chosen with the following properties [28]: elastic modulus E = 7.5 MPa, Poisson ratio ν = 0.01, density ρ = 60 kg/m^3^, and elastic limit of compression σ_EC_ = 0.3 MPa. In this study, we assumed that the outer shell was made from acrylonitrile butadiene styrene (ABS) plastic. The behavior of an outer shell manufactured from ABS plastic was chosen with the following properties [29]: elastic modulus E = 2000 MPa, Poisson ratio ν = 0.37, density ρ = 1200 kg/m^3^, and ultimate tensile stress σ_BT_ = 40 MPa.

To correctly simulate a contact condition between the skull and the liner, a surface‐to‐surface type of contact with a friction coefficient of 0.2 was employed. The same type of contact, but with friction coefficient of 0.4, was used to model the interaction between the outer surface of shell and the rigid surface interface. For the helmet, a bonding between the inner surface of the shell and the outer surface of the liner was applied.

### HIC

There are several criteria for assessing the severity of head injury. These criteria characterize the ability of the human head to withstand the loads during some period of time without causing serious injury. It is known that the severity of head injuries depends on the intensity and duration of the applied pulse. A short-term load is characterized by low-amplitude movements and generates a vibration. The human body can easily withstand such loads, but after some duration, the damage from the impact load becomes crushing. The HIC is one of the most common and reliable criterion to assess TBI severity. Also, HIC can be applied to assess safety related to a car or personal and sport equipment [30]. The physical sense of the criterion is the maximum integral of the deceleration taken on the time interval, during which HIC attains a maximum value. It is defined as follows:

2 $$HIC = \left[ {\frac{1}{{t_{2} - t_{1} }}\int\limits_{{t_{1} }}^{{t_{2} }} {\alpha dt} } \right]^{2.5} \left( {t_{2} - t_{1} } \right),$$

where t_1_ and t_2_ are the initial and final times (in seconds) of the impact, and the acceleration α is measured in G (1G = 9.81 m/s^2^). Observe that HIC values up to 1250 are non-dangerous to human life, HIC values from 1250 to 1500 cause injuries of moderate severity, and HIC values over 1500 cause fatal injuries [30].

### Model Validation

The human finite element head model generated from the MRI data set was validated using the experimental data of a published experiment of frontal impact on a cadaveric head [31]. This cadaveric experiment has been widely recognized by TBI researchers as a standard to validate their finite element head models. In this experiment, the forehead of a seated stationary human cadaver was impacted by a rigid mass of 5.59 kg moving at a constant velocity (9.94 m/s) along the anterior–posterior direction in the mid-sagittal plane. The head was rotated forward at 45° to the Frankfort plane. The input force on the skull was measured during the impact test. The intracranial pressure–time histories at several cerebral locations were recorded by transducers.

**Fig. 2:**
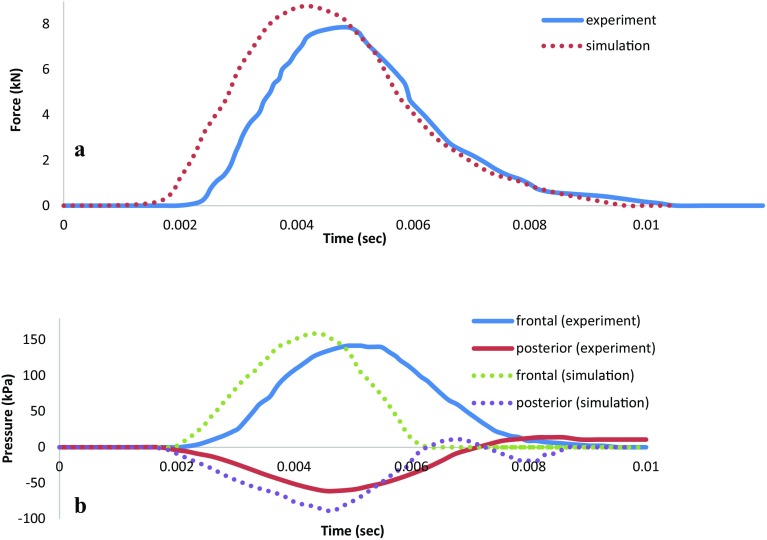
Comparison of impact force (**a**) and intracranial pressure (**b**) between the simulations and the cadaver experiments

To validate human finite element head model, a Nahum’s experiment was numerically replicated. Comparisons between model predictions and experimental measurements of pressure–time histories at coup and contrecoup sites and input force on the skull show good agreement (Fig. 2). The nature of the values of frontal peak pressure, negative peak pressure at posterior fossa and input force on the skull of the simulation were almost the same as those of the Nahum’s experiment, but the numerical values were 10% higher than those from the cadaveric experiment. One or more of the following factors may also contribute to the discrepancy between the model responses and the experimental measurements: exclusion of anatomical structures, such as skin and membranes, simplified models of material behavior, and imprecise information on the exact pressure transducer locations. Therefore, the present finite element head model was considered sufficiently accurate to be used in the following simulations.

## Results

**Fig. 3:**
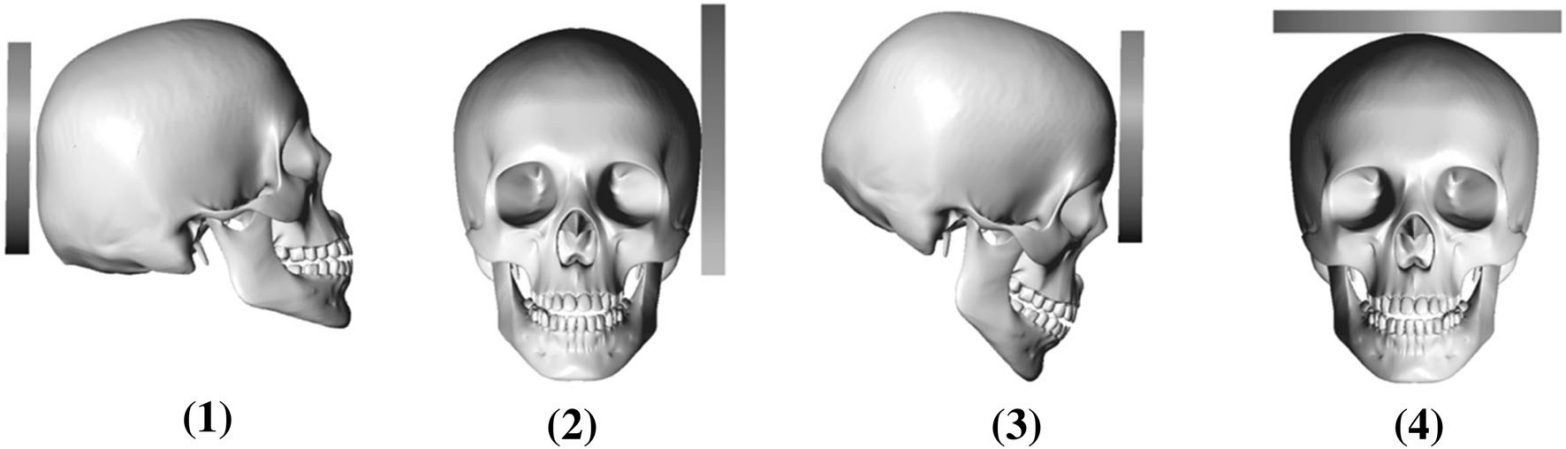
Schematic of impacts to different areas of the head on a hard surface: 1—occiput, 2—temporal, 3—forehead, and 4—parietal

**Fig. 4:**
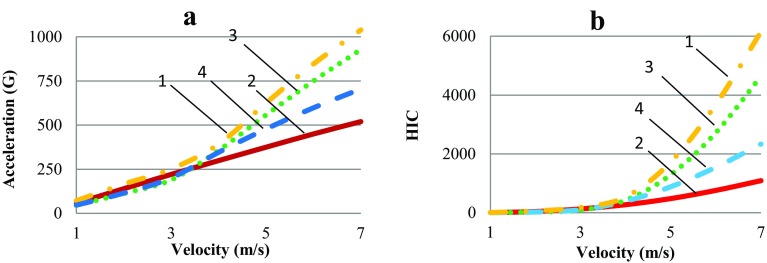
The acceleration value of the center of mass of the head and HIC depending on the velocity of impact. The number of the curve corresponds to the head area of the impact: 1—parietal, 2—occiput, 3—temporal, and 4—forehead

In the first phase of modeling, the data needed to assess the severity of traumatic brain injury that occurs during impact of the head in the cases in which the impact was on the occiput, temporal, forehead, and parietal part and the impact velocity ranged from 1 to 7 m/s were obtained (Fig. 3). The results showed that the severity of damage to the skull and brain upon impact depended on the orientation of the head at the moment of impact, velocity of impact and mechanical properties of the barrier (Fig. 4). The most dangerous impact was on a hard surface (tile, granite, concrete, etc.) because damping of these materials was practically absent; therefore, the time of impact was sharply reduced, increasing the value of the maximum contact force of the impact [32]. This had a negative effect on the severity and extent of damage.

The maximum values of the acceleration occurring at the center of mass of the head in the cases of an impact in different parts with different velocities of impact (Fig. 4) were obtained. HIC is a function that depends on time and on the acceleration of the center of mass of the head; therefore, reducing the impact velocity, all other things being equal, leads to a decrease in HIC values. The results showed that the most dangerous impact was at the parietal and temporal areas of the skull because these areas have the lowest bone thickness of the skull. The obtained HIC values were used to determine the maximum impact speed leading to fatal injuries. In the cases of impacts to the temporal and parietal regions, this speed equaled 5 m/s; in the case of an impact to the forehead region, it equaled 6 m/s; and in the case of an impact to the occiput region, it equaled 7 m/s (Fig. 4b).

To study the effect of a protective helmet in reducing the severity of traumatic brain injury, an impact to the frontal part of the head in the helmet with velocity of 5 m/s was simulated. In the case of choosing the most rational parameters of helmet, the foam thickness was varied from 2 to 20 mm with an increment of 2 mm, and the thickness of the outer protective shell was varied from 1 to 10 mm with an increment of 1 mm.

**Fig. 5:**
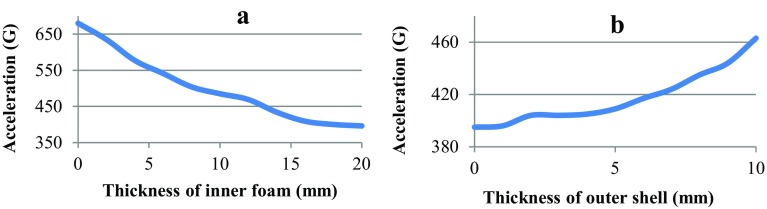
Diagrams characterizing the maximum value of the acceleration of the center of mass of the head depending on the thickness of the inner foam (**a**) and the outer shell (**b**)

The results showed that in the case of increasing the thickness of the foam layer from 0 to 16 mm, a reduction in the magnitude of the acceleration of the center of mass of the brain with a high intensity was achieved (Fig. 5a). In this case, the acceleration was reduced from 635 to 409 G. A further increase in the thickness of the layer of foam on the acceleration of the center of mass of the brain had no effect. Also, obtained data on the acceleration and duration of impact were interpreted in a HIC, which showed that the use of the helmet with a foam thickness of 16 mm reduced the HIC value 3.5 times from 2130 to 600. This indicates that in real life, a 70% probability of severe head injury or 40% probability of fatal injury is reduced to a 90% probability of minor injury [30]. However, in the case of higher impact velocities due to higher contact forces, the thickness of the layer of foam may be not enough, as it could be completely crushed and contact could occur between the head and the traumatic surface.

The results also showed that increasing the thickness of the outer shell has a negative impact on the damping properties of the helmet (Fig. 5b). This occurs because ABS plastic has a high stiffness and high density, but it does not have damping properties. High density is characterized by an increase in the weight of the helmet due to the increased thickness of the outer shell. This leads to even greater values of acceleration of the center of mass of the brain and a greater likelihood of more serious injury. Therefore, in this calculation, the thickness of the outer shell was assumed to be 2 mm, and the results showed that this thickness is enough as it redistributes the force on a broader area of the inner absorbent layer.

**Fig. 6:**
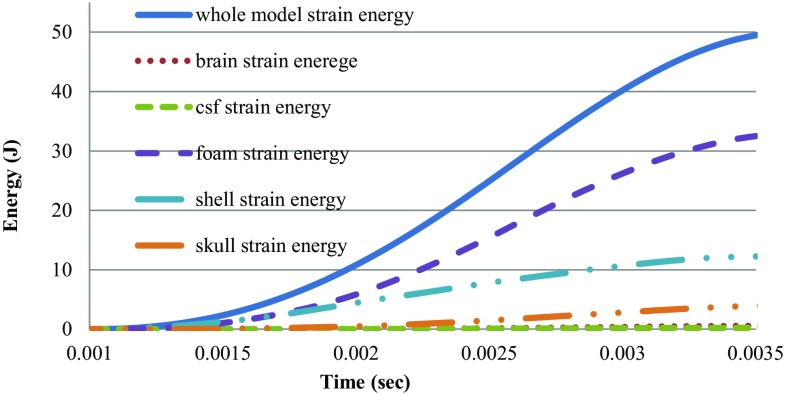
Strain energy in the different parts of head and in the helmet’s outer shell and foam

Figure 6 plots the strain energy absorbed by the foam, shell and parts of head during the impact. It is clearly shown that the kinetic energy was almost completely (90%) transferred into strain energy in the helmet. The initial kinetic energy of the protected head of mass 4.26 kg was approximately 53.2 J (100%). The foam absorbed almost 34.74 J (65.3%) of the energy, whereas the shell absorbed 13 J (24.5%) of the energy. This result indicates that 5.46 J (10.2%) acts on the head and that moreover, only 0.69 J (1.3%) acts on the brain, 4.41 J (8.3%) acts on the skull, and 0.32 J (0.6%) acts on the CSF. In this case, a lower amount of energy is being transferred to the head (in comparison to the unprotected head), and lower corresponding translational acceleration and skull stress levels are experienced.

After the establishment of rational parameters of the protective helmet (2 mm outer hard layer and 16 mm internal damping layer), a detailed simulation of the impact on a hard surface at a speed of 5 m/s to the forehead part of the head in cases with and without helmet was carried out. The purpose of this simulation was biomechanical analysis of the processes that occur in biological tissues during a head impact process.

**Fig. 7:**
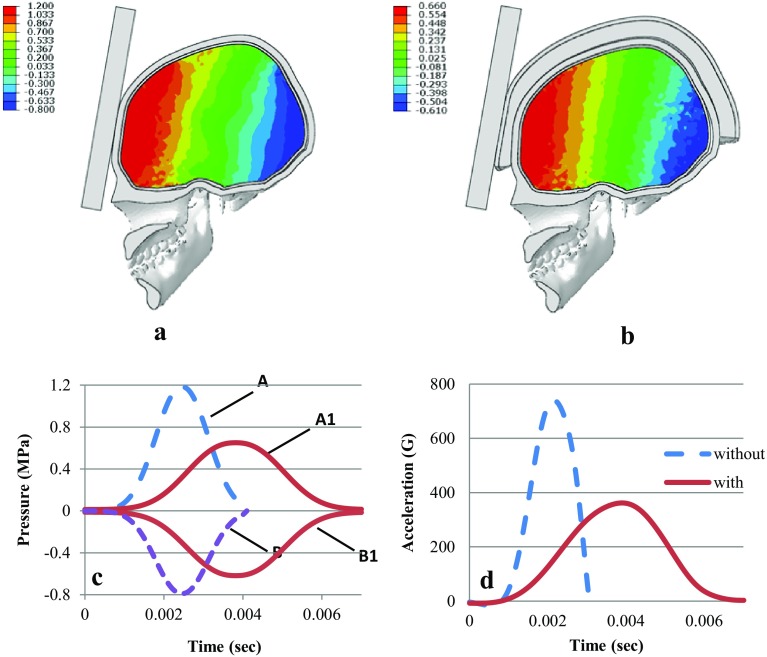
Distributions of the maximum values of pressures in the transversal section of the brain after impact with (**a**) and without helmet (**b**) in MPa. Coup and contrecoup pressure (**c**) and acceleration (**d**) time histories in the cases of a protected and unprotected head from FE simulation

The brain is the most sensitive and injury prone part of the head, and in order to improve our understanding of the characteristics of brain injury during a head impact process, the pressure change in various parts of the brain were examined. Figure 7a, b show the distribution of maximum values of the intracranial pressure in the transversal section of the head during the impact process of unprotected and protected heads on a rigid surface. In this case, the pressure is evenly distributed across the brain with compression in the frontal region and tension in the occipital region. The results showed a maximum compression of 1.2 MPa at the site of impact (coup injury) and a maximum tension of (−0.8) MPa in the opposite area (countercoup injury). In the case of the helmeted head, the values of intracranial pressure at the site of impact and in the opposite area were also obtained. The maximum value of the compressive intracranial pressure, located in the frontal region (the site of impact), was equal to 0.66 MPa, and the negative value of the tensile pressure located in the occipital region was equal to (−0.62 MPa). Figure 7c shows a comparison of intracranial pressures during the impact in the case of a protected and an unprotected model of the human head at the site of impact and in the opposite area. In this figure, the curves correspond to a change in intracranial pressure at the site of impact in the case of an unprotected (A) and a protected (A1) head. Curve B corresponds to the change in the intracranial pressure in the opposite area in the case of an unprotected head helmet, whereas curve B1 represents a protected head helmet.

The acceleration of the center of mass of the protected and unprotected head during impact is shown in Fig. 7d. The duration of impact for the helmeted head was equal to 0.007 s, and it was 0.003 s for the unprotected head. As shown in Fig. 7d, the maximum value of acceleration in the analysis in the first case (protected head) is 50% (361 G), while the results of the analysis of the second case (unprotected head) was 737 G. Also, it should be noted that the decrease in acceleration in the case of the protected head and the increase in the duration of impact reduce the load on the brain compared to unprotected head. In this case, the HIC was reduced 3.5 times from 2130 to 6000. In general, these results demonstrate the excellent quality of the helmet as personal protective equipment for the head.

## Discussion

Studies on mechanical functioning of various biological systems, in particular human organs, allow us to obtain new qualitative characteristics of the biomechanical systems to develop new principles of diagnosis in the early stages of various diseases and to specify the requirements of safety criteria in various areas of human activity. The process of identifying injury mechanisms is comprised of two tasks: first, the field parameter distributions sustained by the tissues in the head under impact conditions are calculated, and second, the link between calculated deformations and observed injuries seen in patients is provided. The proposed finite element model of the head can be used as a tool to explain the different mechanisms of brain damage. The numerical experiment can be employed to diagnose and predict the severity of TBI. In addition, the constructed model can be applied to determine rational parameters of elements of personal protection of the head. In this work, the impact of a 3D model of the human head in the cases when the impact of the head on a surface occurred on different parts of the head and with different impact velocities was studied. The results of numerical simulation confirmed the theory of inertial intracranial displacement of the brain. It found that a positive pressure occurs at the site side of impact, whereas a negative pressure occurs on the opposite side of the skull. The movement of the skull after the impact terminates before the movement of the brain, which moves in the direction of impact and then abruptly returns to the initial position. Appearance of countercoup injury was due to impact of the brain on the inner surface of the skull in the case of its return to the initial position. Furthermore, a method to determine the rational parameters of a protective helmet was developed. The helmet absorbs 90% of the kinetic energy of the system, with the foam absorbing almost 72.5% and the shell absorbing approximately 27.5% of this absorbed energy. The use of a protective helmet reduced the acceleration of the center of mass and the maximum value of intracranial pressure at the site of impact more than twice, and in the opposite area, they were reduced 1.3 times. The HIC value, which characterizes the severity of traumatic brain injury, was reduced 3.5 times from 2130 to 600.

The results of the current study are in accordance with a previous statement from finite element modeling reported by Zhou et al. [33], in which they reported a reduction of acceleration of approximately 60% by using head protection and that the peak value was reduced by 57.7% compared to that of the unprotected head at a thickness of 10 mm. Cripton et al. [34] conducted an experiment with biomechanical testing of helmeted and unprotected head impacts using a validated anthropomorphic test headform and a range of drop heights between 0.5 and 3.0 m while measuring headform acceleration and HIC. The results proved that contemporary bike helmets are highly effective at reducing injury risk through paired helmeted and unprotected impacts with realistic drop heights and impact speeds. For example, he showed that the helmets reduced the head peak acceleration from 824 to 181 g for drops of 2.0 m, reducing the risk of skull fracture from 99.9 to 5%.

We also compared this model with Total Human Model for Safety (THUMS) and Wayne State University Head Injury Model (WSUHIM). THUMS, which includes the whole body, has been used for the detailed investigation of interactions between human bodies and vehicular structures, including some safety devices, precrash and during a crash. Wayne State University Head Injury Model has been used in impact biomechanics studies of automotive crashes, sports injuries, blast injury, etc. These models (THUMS and WSUHIM) include the scalp, a three-layered skull, cerebrospinal fluid, dura mater, falx cerebri, and brain with differentiated white and gray matter. Both models have been validated using published cadaveric test data reported by Nahum et al. [31] and Trosseille et al. [35] on intracranial and ventricular pressure; Hardy et al. [36, 37] and King et al. [38] on relative displacement between the brain and the skull; and Nyquist et al. [39] and Allsop et al. [40] upon facial impact.

However, validation of our model using published cadaveric test data reported by Nahum et al. showed a close agreement between the results. Thus, this indicated that the number of the individual anatomical structures in the head model does not play a significant role in the numerical accuracy of the head model as long as the major structures have been created.

In summary, it should be noted that finite element analysis is a highly precise numerical method of analysis that enables the study of stress distribution in biological systems. However, our study has some limitations within which our findings need to be interpreted carefully. First, we used a simple material model of skull bone. In a realistic situation, bone is a complex heterogeneous structure. Second, we did not consider the scalp, cervical vertebrae, or intervertebral discs, and the brain was not separated into white matter and gray matter. Third, the size of the head should have an influence on the results. However, a change in head size is also associated with an increase/decrease in the skull thickness and/or CSF volume, which plays a significant role in brain protection. This indicates that it is impossible to predict the influence of head size on the results of the impact model without further investigation, and thus more thorough research is needed. The results presented in this study are associated with the average head size of a European male.

## Conclusion

A detailed finite element model of head was proposed and validated in a frontal impact configuration. This model was used to investigate the brain injury level when the head impacts came from different directions with different velocities. Also, two analyses were carried out in which the impact of a protected head and that of an unprotected head were simulated. In the case of the protected head, rational helmet parameters were determined. Various parameters, such as the duration of impact, intracranial pressure, HIC, and change of accelerations of the center of mass of brain were determined and compared. The highest brain acceleration was observed for the temporal and parietal impacts, signifying that these impacts are the most severe cases of head impact. The directional dependence of the head response was confirmed, as it was observed that for temporal and parietal impacts, the head was less prone to severe injuries, while temporal and parietal impacts predicted severe injury conditions. In the case of searching for efficient parameters of the protective helmet model, the foam thickness was shown to be the most important influence on HIC response, even more than the shell. The comparison of the analysis results showed significant differences in severity of head injury between the protected and unprotected head. Thus, the proposed finite element model allowed us to predict the severity of TBI in protected or unprotected human heads and to provide recommendations for determining the rational parameters of different types of helmets.
